# Fluorodeoxyglucose versus Choline Positron Emission Tomography/Computed Tomography Response Evaluation in Two Malignant Pleural Mesothelioma Patients Treated with Talc Pleurodesis and Neoadjuvant Chemotherapy

**DOI:** 10.7759/cureus.3654

**Published:** 2018-11-28

**Authors:** Kazuhiro Kitajima, Toru Nakamichi, Seiki Hasegawa, Kozo Kuribayashi, Koichiro Yamakado

**Affiliations:** 1 Radiology, Hyogo College of Medicine, Nishinomiya, JPN; 2 Thoracic Surgery, Hyogo College of Medicine, Nishinomiya, JPN; 3 Internal Medicine, Hyogo College of Medicine, Nishinomiya, JPN

**Keywords:** malignant pleural mesothelioma, treatment response, pet/ct, fdg, choline

## Abstract

Talc pleurodesis has been reported to increase fluorodeoxyglucose (FDG) uptake in the high attenuation areas of pleural thickening, making it difficult to distinguish between benign granulomatous inflammatory processes and malignancies, which may therefore interfere with the post-chemotherapy disease evaluation on FDG-positron emission tomography/computed tomography (PET/CT). We present two cases of malignant pleural mesothelioma treated with talc pleurodesis and neoadjuvant chemotherapy (NAC) before pleurectomy/decortication in which post-NAC FDG-PET/CT showed intense FDG uptakes in the high attenuation areas of pleural thickening with false positive result, whereas post-NAC ^11^C-choline PET/CT showed mild choline uptake of pleural talc deposit, which did not interfere with the post-chemotherapy disease evaluation. Thus we suggest choline-PET/CT may show little choline uptake to granulomatous inflammation and evaluate treatment response in malignant pleural mesothelioma patients treated with talc pleurodesis and NAC.

## Introduction

Positron emission tomography/computed tomography (PET/CT) with fluorodeoxyglucose (FDG) has been a useful tool to manage patients with malignant pleural mesothelioma in the several clinical situations of diagnosis, initial staging, therapy planning, treatment response assessment, re-staging, and prognostication [1]. However, talc pleurodesis has been reported to increase FDG uptake in the high attenuation areas of pleural thickening, making it difficult to distinguish between benign granulomatous inflammatory processes and malignancies, with the potential for false positive results on FDG-PET/CT scans [2-6]. Therefore, talc pleurodesis may interfere with the post-chemotherapy disease evaluation on FDG-PET/CT. A new positron emission tomography (PET) tracer that can decrease uptake to granulomatous inflammation and evaluate treatment response in malignant pleural mesothelioma patients treated with talc pleurodesis and neoadjuvant chemotherapy (NAC) could be of tremendous value.

Choline uptake may occur via a choline-speciﬁc transporter protein and could be accelerated during tumor cell proliferation on choline-PET/CT. Here we report two cases of malignant pleural mesothelioma treated with talc pleurodesis and NAC before pleurectomy/decortication in which post-NAC FDG-PET/CT showed a false-positive result due to talc pleurodesis, whereas post-NAC choline-PET/CT showed faint choline uptake of pleural talc deposit, which did not interfere with the post-chemotherapy disease evaluation.

## Case presentation

Patient 1

**Figure 1:**
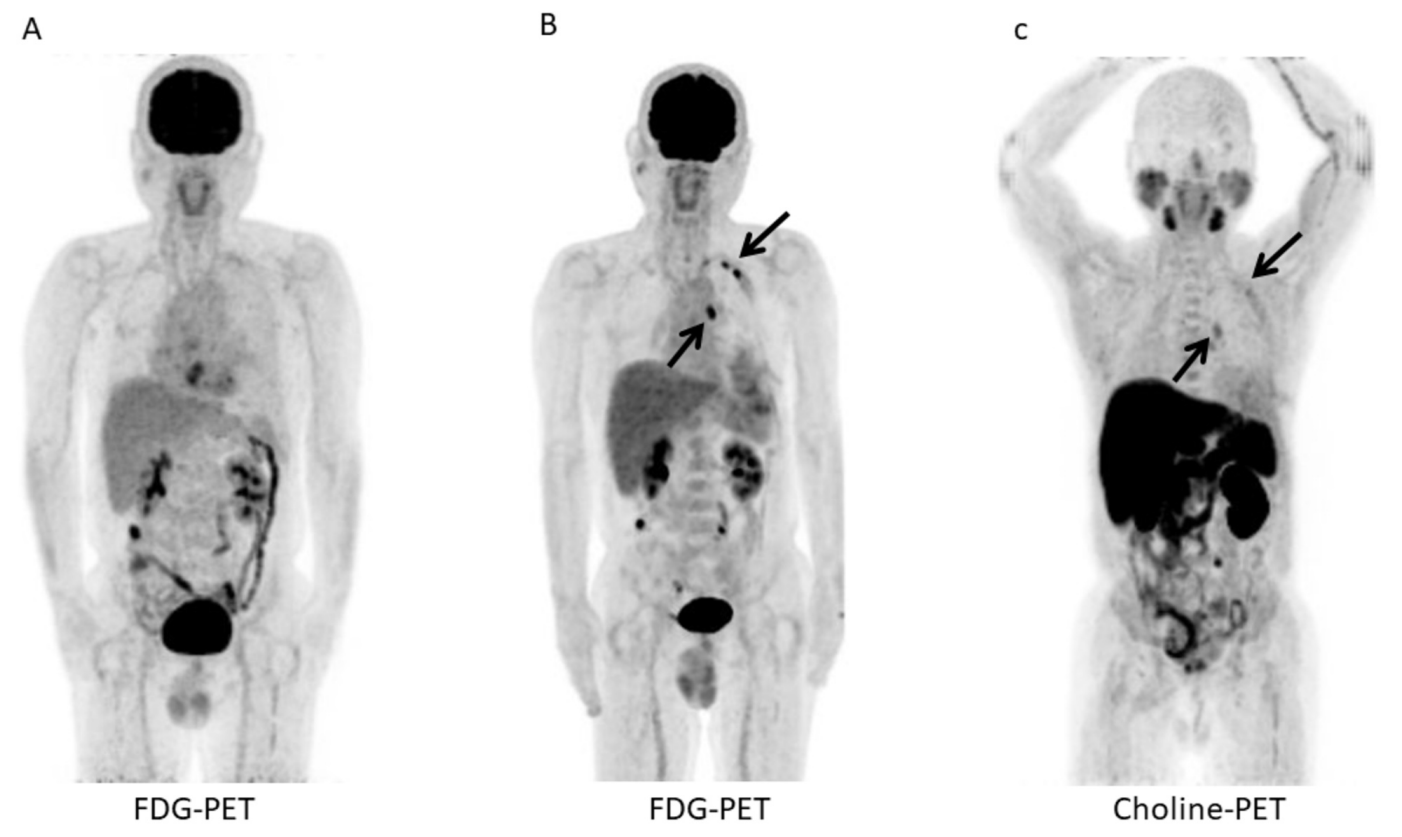
Fluorodeoxyglucose (FDG)-positron emission tomography/computed tomography (PET/CT) before and after talc pleurodesis and neoadjuvant chemotherapy (NAC) and 11C-choline PET/CT after talc pleurodesis and NAC for patient 1. A. Maximum intensity projection (MIP) of fluorodeoxyglucose (FDG)-positron emission tomography/computed tomography (PET/CT) before talc pleurodesis and neoadjuvant chemotherapy (NAC) shows no abnormal FDG uptake of the left pleura. B. MIP of FDG-PET/CT after talc pleurodesis and NAC shows three abnormal FDG uptakes of the left pleura (arrows). C. MIP of 11C-choline PET/CT after talc pleurodesis and NAC shows faint choline uptakes of the left pleura (arrows).

**Figure 2:**
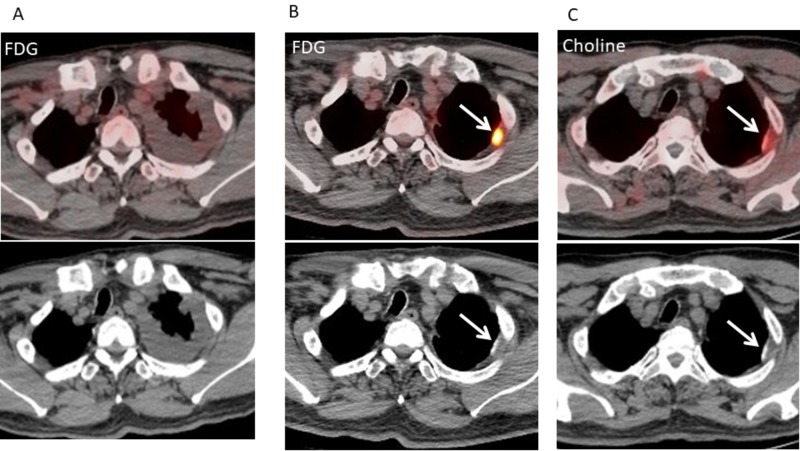
Fluorodeoxyglucose (FDG)-positron emission tomography/computed tomography (PET/CT) before and after talc pleurodesis and neoadjuvant chemotherapy (NAC) and 11C-choline PET/CT after talc pleurodesis and NAC for patient 1. A. Axial fluorodeoxyglucose (FDG)-positron emission tomography/computed tomography (PET/CT) before talc pleurodesis and neoadjuvant chemotherapy (NAC) shows left pleural effusion and no abnormal FDG uptake of the left pleura. B. Axial FDG-PET/CT after talc pleurodesis and NAC shows intense FDG uptake (maximum standardized uptake value, 5.53) in the high attenuation area of left pleural thickening (arrow). C. Axial 11C-choline PET/CT after talc pleurodesis and NAC shows mild choline uptake (maximum standardized uptake value, 2.75) in the high attenuation area of left pleural thickening (arrow).

A 68-year-old man visited our hospital due to exertional dyspnea. His past medical history revealed diabetes mellitus, hypertension, and benign prostate hyperplasia. He had worked as a building housebreaker and at a processing company for plastic and had been exposed to asbestos. Cytological examination of the pleural effusion and pleural biopsy during talc pleurodesis yielded a diagnosis of epithelial malignant pleural mesothelioma. Immunohistochemical analyses demonstrated that these cells were positive for AE1/AE3, calretinin, D2-40, WT-1, mesothelin, HEG1, CD146, EMA, MTAP, p16, and p53, and negative for CEA, TTF-1, desmin, and BAP1 and Ki67 index was 5%. FDG-PET/CT before talc pleurodesis showed left pleural effusion and no FDG uptake of the left pleura (Figures 1A, 2A). He did not undergo 11C-choline PET/CT scan before pleurodesis and NAC. He underwent NAC of three courses of cisplatin and pemetrexed. FDG-PET/CT after talc pleurodesis and NAC showed intense FDG uptakes in the high attenuation areas of left pleural thickening (Figures 1B, 2B), whereas 11C-choline PET/CT showed mild choline uptake of left pleural talc deposit (Figures 1C, 2C). Although it is difficult to evaluate treatment response of NAC due to a false-positive result by FDG-PET/CT, choline-PET/CT did not interfere with the post-chemotherapy disease evaluation. Pleurectomy/decortication was performed. The disease was categorized as T3N1M0 and mild treatment response was observed (grade 1a). He received adjuvant chemotherapy consisting carboplatin and pemetrexed and remains well 10 months after the definite diagnosis.

Patient 2

**Figure 3:**
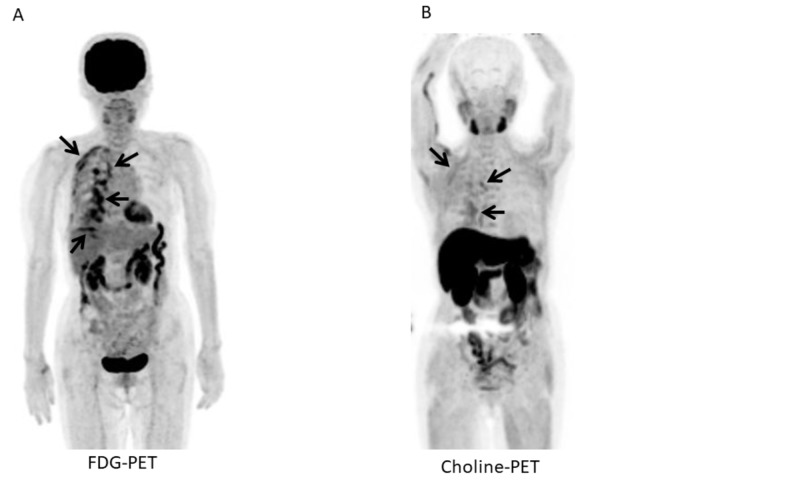
Fluorodeoxyglucose (FDG) and 11C-choline positron emission tomography/computed tomography (PET/CT) after talc pleurodesis and neoadjuvant chemotherapy (NAC) for patient 2. A. Maximum intensity projection (MIP) of fluorodeoxyglucose (FDG)-positron emission tomography/computed tomography (PET/CT) after talc pleurodesis and neoadjuvant chemotherapy (NAC) shows many abnormal FDG uptakes of the right pleura (arrows). B. MIP of 11C-choline PET/CT after talc pleurodesis and NAC shows faint choline uptakes of the right pleura (arrows).

**Figure 4:**
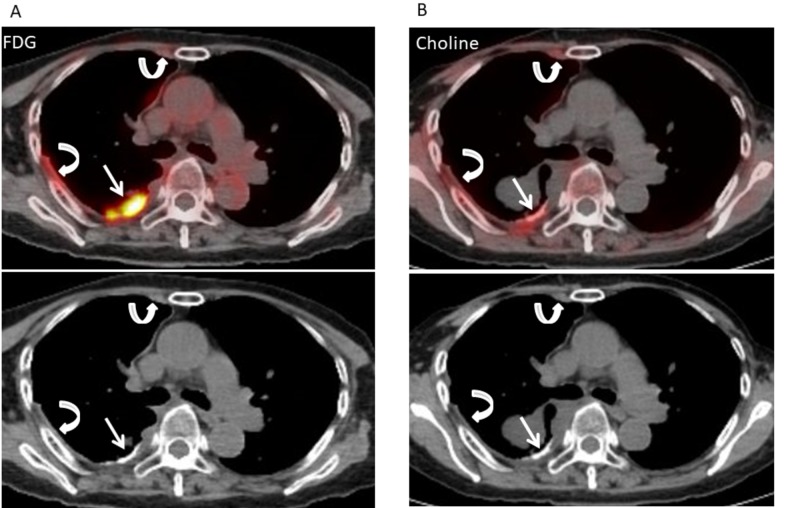
Fluorodeoxyglucose (FDG) and 11C-choline positron emission tomography/computed tomography (PET/CT) after talc pleurodesis and neoadjuvant chemotherapy (NAC) for patient 2. A. Axial fluorodeoxyglucose (FDG)-positron emission tomography/computed tomography (PET/CT) after talc pleurodesis and NAC shows intense FDG uptake (maximum standardized uptake value, 5.74) in the high attenuation areas of right pleural thickening (arrow) and mild FDG uptakes (maximum standardized uptake value, 2.47) in the non-high attenuation areas of right pleural thickening (arrows). B. Axial 11C-choline PET/CT after talc pleurodesis and NAC shows mild choline uptake (maximum standardized uptake value, 2.97) in the high attenuation areas of right pleural thickening (arrow) and faint choline uptakes (maximum standardized uptake value, 2.19) in the non-high attenuation areas of right pleural thickening (arrows).

A 77-year-old female visited our hospital for examination of right pleural effusion. She had undergone the operation of bilateral breast cancers and sigmoid cancer in the past. She had not been exposed to asbestos. Cytological examination of the pleural effusion and pleural biopsy during talc pleurodesis yielded a diagnosis of epithelial malignant pleural mesothelioma. Immunohistochemical analyses demonstrated that these cells were positive for AE1/AE3, calretinin, D2-40, WT-1, mesothelin, HEG1, CD146, MTAP, and p53, and negative for CEA, TTF-1, and claudin-4 and Ki67 index was 8%. She did not undergo FDG-PET/CT and 11C-choline PET/CT scans before pleurodesis and NAC. She underwent NAC of three courses of cisplatin and pemetrexed. FDG-PET/CT after talc pleurodesis and NAC showed intense FDG uptakes in the high attenuation areas of right pleural thickening (Figures 3A, 4A), whereas 11C-choline PET/CT showed mild choline uptake of right pleural talc deposit (Figures 3B, 4B). Although it is difficult to evaluate treatment response of NAC due to false-positive result by FDG-PET/CT, choline PET/CT did not interfere with the post-chemotherapy disease evaluation. Pleurectomy/decortication was performed. The disease was categorized as T3N1M0 and mild treatment response was observed (grade 1a). She received adjuvant chemotherapy consisting carboplatin and pemetrexed and remains well seven months after the definite diagnosis.

## Discussion

Talc pleurodesis is widely performed in the management of malignant pleural mesothelioma patients with persistent pleural effusion. The pleural inflammatory response to talc administration is considered to be responsible for its ability to cause pleurodesis. Pathologic studies have indeed demonstrated a strong inflammatory reaction of both the visceral and parietal pleura following pleural talc administration, leading to the formation of pleural talc granulomata and, eventually, pleural fibrosis [7].

A first case report on FDG uptake after talc pleurodesis has been previously published [2]. It described increased FDG accumulation that corresponded to high density plaques on computed tomography (CT). The findings were attributed to talc pleurodesis. These findings of glucose hypermetabolism should be interpreted with caution and not simply misdiagnosed as malignancy. The ability of PET/CT to fuse and correlate FDG and CT findings may be helpful in differentiating malignant from talc-induced granulomatous lesions by providing the exact CT morphologic feature that matches the tracer accumulation. Allocation of hypermetabolic foci to lesions of increased density should lead to the estimated diagnosis of talc-induced granulomatous lesions in the presence of respective history. However, the core problem of misleading FDG accumulation in granulomatous tissue cannot be solved by the use of PET/CT hybrid imaging devices, and talc pleurodesis may underestimate the tumor response to chemotherapy on FDG-PET/CT. A new PET tracer that can decrease uptake to granulomatous inflammation and evaluate treatment response in malignant pleural mesothelioma patients treated with talc pleurodesis and NAC could be of tremendous value.

To our knowledge, two groups have evaluated the diagnostic performance of FDG-PET/CT for evaluating treatment response in malignant pleural mesothelioma patients treated with talc pleurodesis and NAC. Pillong et al. [8] demonstrated that 16 of the 24 FDG-PET/CTs in 20 malignant pleural mesothelioma patients were performed following talc pleurodesis and talc pleurodesis made no significant difference to the accuracy of intrathoracic staging. Genestreti et al. [6] demonstrated that the metabolic response measured by mean standardized uptake value (SUVmean) seems to be in better agreement with the radiologic response compared to the maximum SUV (SUVmax) in eight malignant pleural mesothelioma patients treated with talc pleurodesis and NAC.

11C- and 18F- choline PET/CT has been successfully used for prostate cancer restaging in patients with biochemical disease recurrence after deﬁnitive therapy and staging advanced prostate cancer [9]. 11C-choline uptake may occur via a choline-speciﬁc transporter protein that is overexpressed in the membranes of prostate cancer cells [10]. 11C-choline is phosphorylated by choline kinase, which is upregulated and retained within tumor cells for synthesis of phosphatidylcholine. Phosphatidylcholine is an essential component of cell membranes, involved in the modulation of transmembrane signaling during carcinogenesis. Therefore, 11C-choline uptake is accelerated during cancer cell proliferation. To our knowledge, there have been no reports to evaluate malignant pleural mesothelioma using 11C-choline PET/CT and this is the first report to evaluate the treatment response of pleurodesis and NAC in malignant pleural mesothelioma patients.

## Conclusions

It is difficult to evaluate treatment response in malignant pleural mesothelioma patients treated with talc pleurodesis and NAC by FDG-PET/CT. Although choline PET/CT may show little choline uptake to granulomatous inflammation and evaluate treatment response in malignant pleural mesothelioma patients treated with talc pleurodesis and NAC, the clinical usefulness of choline PET/CT for evaluating treatment response in malignant pleural mesothelioma patients treated with talc pleurodesis and NAC has still to be assessed in larger prospective studies.

## References

[1] Kitajima K, Doi H, Kuribayashi K (2016). Present and future roles of FDG-PET/CT imaging in the management of malignant pleural mesothelioma. Jpn J Radiol.

[2] Murray JG, Erasmus JJ, Bahtiarian EA, Goodman PC (1997). Talc pleurodesis simulating pleural metastases on 18F-fluorodeoxyglucose positron emission tomography. AJR Am J Roentgenol.

[3] Kwek BH, Aquino SL, Fischman AJ (2004). Fluorodeoxyglucose positron emission tomography and CT after talc pleurodesis. Chest.

[4] Ahmadzadehfar H, Palmedo H, Strunk H, Biersack HJ, Habibi E, Ezziddin S (2007). False positive 18F-FDG-PET/CT in a patient after talc pleurodesis. Lung Cancer.

[5] Peek H, van der Bruggen W, Limonard G (2009). Pleural FDG uptake more than a decade after talc pleurodesis. Case Rep Med.

[6] Genestreti G, Moretti A, Piciucchi S (2012). FDG PET/CT response evaluation in malignant pleural mesothelioma patients treated with talc pleurodesis and chemotherapy. J Cancer.

[7] Kennedy L, Sahn SA (1994). Talc pleurodesis for the treatment of pneumothorax and pleural effusion. Chest.

[8] Pilling J, Dartnell JA, Lang-Lazdunski L (2010). Integrated positron emission tomography-computed tomography does not accurately stage intrathoracic disease of patients undergoing trimodality therapy for malignant pleural mesothelioma. Thorac cardiov surg.

[9] Kitajima K, Murphy RC, Nathan MA, Sugimura K (2014). Update on positron emission tomography for imaging of prostate cancer. Int J Urol.

[10] Uchida T, Yamashita S (1992). Molecular cloning, characterization, and expression in Escherichia coli of a cDNA encoding mammalian choline kinase. J Biol Chem.

